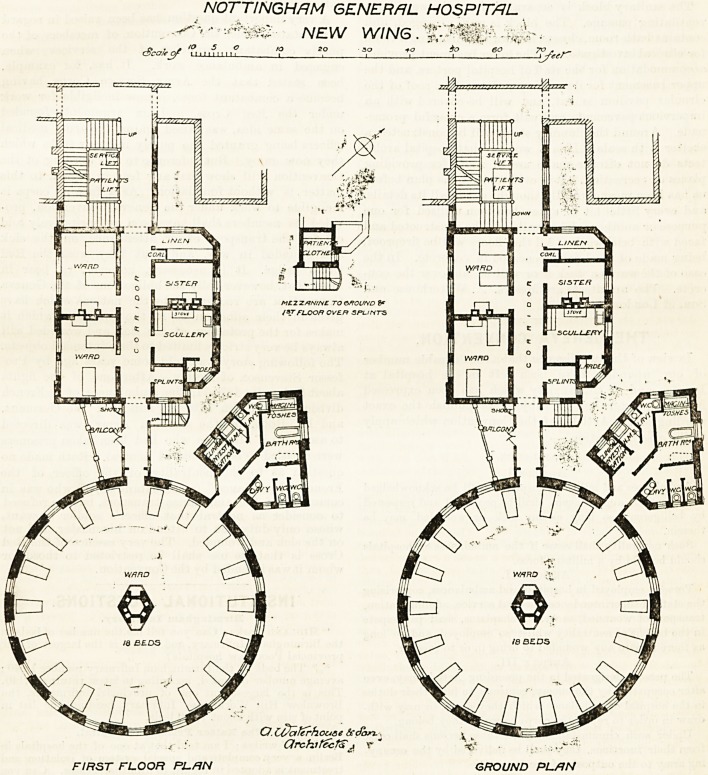# Hospital Construction

**Published:** 1899-10-28

**Authors:** 


### The Institutional Workshop.

### HOSPITAL CONSTRUCTION.

### NOTTINGHAM GENERAL HOSPITAL—JUBILEE WING.

In addition to the basement, this wing is a four storey block connected with the old building by a corridor 81 ft. in length, which joins the present corridor at right angles and extends in a south-easterly direction along the Postern Street frontage. On the right of the corridor of communication are placed two small wards intended for two beds each, and on the left are the linen-room, sisters'-room, scullery, larder, and room for surgical appliances. There is a fine staircase close to the old corridor, and it includes a lift large enough to take a patient in the recumbent position. The main features, however, of the new block are its large circular wards. The pavilion is joined to the wards already described by bridges 16 ft. long. On one side of this is a large balcony, in which beds might be placed, and on the other an emergency staircase. The circular wards are 55 ft. 6 in. in diameter. There are 18 beds, and each bed has 1,800 cubic feet of air space and a window on each side, except in those places where window spaces are occupied by corridors. The chimney shaft rises up the centre of the ward, and is so arranged that it will serve for ventilation as well as a smoke flue. The warming is done by hot-water radiators; but all the wards have open fireplaces also. We are pleased to note this. The fresh air is brought into the wards through the radiators, so that it is warmed at the moment of entrance; and the extract ventilator communicates with the central smoke shaft.

The sanitary block is separated from the ward by a ventilating passage. The block is well arranged, and contains bath-room, closets, lavatories, sinks, and a room for clinical investigation. The lower basement provides accommodation for the staff of hospital porters, and the upper basement for domestic servants. The roof of the circular pavilion is flat, and will be covered with an impervious pavement, and will form a cheerful promenade. Around the chimney stack will be constructed a shelter with seats. It is a wonder that hospital architects do not oftener make use of roofs for providing places of recreation. It is evident that the plan before us has been most carefully thought out in all its details, and every little bit of space has been utilised for one purpose or another. The walls will be constructed and faced with brickwork. All the floors will be fireproof, being made of steel joists encased in concrete. In the case of the wards a teak floor will be laid over the concrete. The architects are Messrs. A. Waterhouse and Son, of London.

## Figures and Tables

**Figure f1:**